# Sex-specific patterns and limited bilateral symmetry in coronal knee alignment: Insights from the modified coronal plane alignment of the knee classification

**DOI:** 10.1016/j.ocarto.2025.100721

**Published:** 2025-11-28

**Authors:** Kyota Ishibashi, Ryoto Kura, Eiji Sasaki, Hikaru Kristi Ishibashi, Yuka Kimura, Yukiko Sakamoto, Eiichi Tsuda, Yasuyuki Ishibashi

**Affiliations:** a Department of Orthopaedic Surgery, Hirosaki University Graduate School of Medicine, Hirosaki, Japan; b Department of Rehabilitation Medicine, Hirosaki University Graduate School of Medicine, Hirosaki, Japan

**Keywords:** Knee joint, Osteoarthritis, Knee, Sex characteristics, Symmetry, Radiography

## Abstract

**Objective:**

The modified Coronal Plane Alignment of the Knee (CPAK) classification broadens the neutral boundaries of arithmetic hip–knee–ankle angle (aHKA) and introduces the concept of arithmetic joint line obliquity (aJLO). This study aimed to compare the original and modified CPAK classifications and to evaluate their impact on sex-specific distribution and bilateral asymmetry.

**Design:**

A total of 673 adults (400 women) from the Iwaki Health Promotion Project were analyzed. The aHKA was calculated as follows: aHKA = medial proximal tibial angle (MPTA) – lateral distal femoral angle (LDFA). The aJLO was calculated as follows: 90° – (LDFA + MPTA)/2. The original CPAK classification categorized knees into nine phenotypes using the aHKA and JLO, with neutral boundaries defined as 0° ± 2°. The modified CPAK classification adopted a wider neutral boundary of 0° ± 3° for the aHKA and aJLO. Sex-stratified analyses and bilateral comparisons were conducted.

**Results:**

The modified CPAK classification redistributed the phenotypes more evenly and increased the proportion of neutral–neutral type V knees to 23.2% (vs. only 3.1% with the original CPAK classification). Men more frequently showed varus phenotypes, particularly type I (20.8%), whereas women had a higher prevalence of neutral (25.8%) and valgus phenotypes. Bilateral knee analysis revealed strong correlations with aHKA (*r* ​= ​0.71, *p* ​< ​0.001) and aJLO (*r* ​= ​0.73, *p* ​< ​0.001). However, only 58% of individuals showed identical CPAK types bilaterally.

**Conclusions:**

Compared with the original system, the modified CPAK classification offers a more balanced representation of coronal alignment in Asian knees.

## 1 Introduction

Coronal alignment of knees plays a pivotal role in both knee osteoarthritis (OA) development and knee arthroplasty outcomes [[1], [2], [3], [4], [5]]. With the shift in surgical concepts from mechanical alignment toward kinematic or phenotype-oriented alignment strategies, precise characterization of constitutional alignment has become increasingly critical for both epidemiological research and surgical planning.

Recent studies on functional knee phenotypes have demonstrated wide inter-individual variability in femoral and tibial alignment, showing that even non-OA knees display diverse coronal morphologies and comparable joint line orientations between OA and non-OA populations [[6], [7], [8]]. These findings emphasize the complexity of native alignment and the need for refined classification systems that can capture such variability.

The Coronal Plane Alignment of the Knee (CPAK) classification, introduced by MacDessi et al., in 2021, provides a comprehensive yet practical system for categorizing knee phenotypes into nine types [9]. Nevertheless, the original CPAK classification reportedly tends to cluster disproportionately into apex-distal types in Asian populations, who typically exhibit greater constitutional varus, consequently underrepresenting the neutral phenotypes [[10], [11], [12], [13]]. To address this limitation, Hsu et al. proposed the modified CPAK classification, which broadens the neutral boundaries of the arithmetic hip–knee–ankle angle (aHKA) (0° ​± ​3° instead of ±2°) and introduces the concept of arithmetic joint line obliquity (aJLO), representing the true joint line angle relative to the ground [14]. Despite these refinements, several important knowledge gaps remain. First, although sex-related differences in knee alignment are well-established, differences in CPAK migration patterns between men and women have not yet been evaluated. Second, coronal plane alignment is often assumed to be symmetrical between limbs; however, epidemiological data on bilateral CPAK concordance versus asymmetry within individuals remain scarce.

Therefore, the present study aimed to investigate the CPAK classification in a Japanese population by (i) comparing the distribution of knee types between the original and modified CPAK classifications, (ii) analyzing classification migration patterns stratified by sex and knee OA severity, and (iii) evaluating the frequency and patterns of bilateral asymmetry. This study is expected to provide new insights into the applicability of CPAK classification in Asian populations and to highlight the clinical implications of sex-specific and side-to-side alignment differences.

## 2 Methods

### 2.1 Participants

All participants were volunteers of the Iwaki Health Promotion Project, a community-based preventive medicine program aimed at improving average life expectancy through general health checkups and prophylactic interventions [[15], [16], [17], [18], [19]]. This study was approved by the Ethics Committee of Hirosaki University Graduate School of Medicine (approval no. 2021-019H_7) and was conducted in accordance with the tenets of the 1964 Helsinki Declaration and its later amendments.

In total, 737 volunteers (430 women) participated in the Iwaki Health Promotion Project in 2022. Among them, participants who had a history of hip or knee arthroplasty (*n* ​= ​3), were diagnosed with rheumatoid arthritis (*n* ​= ​16), had a history of lower-limb fractures (*n* ​= ​29), had previously undergone knee surgeries (*n* ​= ​12), and did not undergo radiography (*n* ​= ​4) were excluded from the analyses. Finally, 673 participants (400 women) were analyzed in this study.

### 2.2 Knee phenotypes

Standing digital long-leg radiographs were obtained from all participants, with both patellae directed forward and knees fully extended. Radiographs were acquired using a digital radiography system (CXDI-40EG, Canon Inc., Tokyo, Japan) under the following standardized conditions: cassette-to-tube distance of 300 ​cm, tube voltage of 85 ​kV, and tube current of 200 ​mA. During imaging, the participants stood barefoot with their legs closed and patellae facing forward.

Lower-limb alignment was radiographically measured using mediCAD® software version 5.5 (TOYO Corporation, Tokyo, Japan) [20]. The hip–knee–ankle angle (HKAA), mechanical lateral distal femoral angle (mLDFA), femoral mechanical axis, medial proximal tibial angle (MPTA), and joint line convergence angle (JLCA) were assessed. The HKAA was defined as the angle between the mechanical femoral and tibial axes. The mLDFA referred to the lateral angle between the femoral mechanical axis and distal femoral joint line. The MPTA was defined as the medial angle between the tibial mechanical axis and proximal tibial joint line. The JLCA referred to the angle between the distal femoral and proximal tibial joint surfaces, with a positive JLCA value indicating openings [13].

Functional knee phenotypes for the lower limb, femur, and tibia were characterized based on their respective alignment angles—HKAA, FMA, and MPTA [21]. Each phenotype was defined by a phenotype-specific mean value with a tolerance range of ±1.5°, corresponding to 3° increments between adjacent phenotypes. The nomenclature followed the convention of describing alignment direction (Neutral: NEU, Varus: VAR, Valgus: VAL), measurement parameter (HKAA, FMA, MPTA), and mean deviation (0°, 3°, or 6°). For instance, the phenotype “NEU_HKA_0° ​+ ​NEU_FMA_0° ​+ ​NEU_MPTA_0°” represents a neutral limb, femoral, and tibial alignment. Functional knee phenotypes were then determined as the combination of limb, femoral, and tibial phenotypes, enabling a comprehensive characterization of coronal alignment. Considering five phenotypes for each component (VAL6°, VAL3°, NEU0°, VAR3°, VAR6°), a total of 125 combinations were theoretically possible.

Inter-observer agreement was assessed using intraclass correlation coefficients to evaluate measurement reliability. Two experienced senior orthopedic surgeons independently measured 50 radiographs of 100 knees. ICC (2,1) values indicated excellent reproducibility for all parameters, with coefficients being consistently greater than 0.75 (range: 0.87–0.99). Specifically, the intraclass correlation coefficients were 0.998 (0.997–0.999) for the HKAA, 0.992 (0.985–0.995) for the mLDFA, 0.953 (0.918–0.972) for the MPTA, and 0.870 (0.775–0.924) for the JLCA.

### 2.3 CPAK classification

Each knee was classified according to both the original CPAK classification introduced by MacDessi et al. and the modified CPAK classification proposed by Hsu et al. [9,14]. The aHKA was calculated as follows: aHKA ​= ​MPTA – mLDFA. The aJLO was calculated as follows: 90° – (LDFA ​+ ​MPTA)/2. The original CPAK classification categorized the knees into nine phenotypes using the aHKA and JLO, with neutral boundaries defined as 0° ​± ​2°. In contrast, the modified CPAK classification adopted a wider neutral boundary of 0° ​± ​3° for the aHKA and introduces the aJLO to more accurately reflect joint line orientation to the ground. Phenotypes were compared between the original and modified CPAK classifications in our cohort.

### 2.4 Severity of knee osteoarthritis

The severity of OA in each knee was classified by two trained orthopedic surgeons (RK and ES) as Kellgren–Lawrence (KL) grade 0 to 4 [22]. The surgeons were blinded to the sequence in which the radiographs were acquired and the clinical status of the participants. For subgroup analysis, participants were categorized into three groups according to KL grade (KL 0–1, KL 2, and KL 3–4), and the distribution of CPAK types was compared among these groups.

### 2.5 Statistical analysis

Categorical and continuous variables were expressed as frequencies with percentages and as means ​± ​standard deviations, respectively. Groups were compared using the Mann–Whitney *U* test. Correlation between continuous variables was examined using Spearman’s correlation test. All analyses were performed using SPSS version 29.0 (IBM Corp., Armonk, NY, USA). Statistical significance was set at *p* ​< ​0.05.

## 3 Results

In total, 673 adults (400 women; mean age: 52.6 ​± ​15.0 years) were included in the present analysis. With respect to alignment parameters, men showed higher HKAA (2.68° ​± ​2.4° vs. 1.95° ​± ​2.6°, *p* ​< ​0.001) and lower MPTA (85.4° ​± ​2.0° vs. 86.3° ​± ​1.9°, *p* ​< ​0.001), whereas women exhibited slightly lower mLDFA (86.6° ​± ​1.9° vs. 87.0° ​± ​1.7°, *p* ​= ​0.009) and higher JLCA (1.55° ​± ​1.5° vs. 1.10° ​± ​1.3°, *p* ​< ​0.001) (Table 1). The ten commonest knee phenotypes among male and female participants are listed in Table 2.

**Table 1:** Clinical characteristics and radiographic parameters of the participants.

	Total	Men	Women	*p*-value
Number	673	273	400	–
Age, years	52.6 ​± ​15.0	53.8 ​± ​15.0	51.8 ​± ​14.9	0.094
Body mass index, kg/m^2^	23.0 ​± ​3.3	24.0 ​± ​3.1	22.4 ​± ​3.3	<0.001
HKAA, degrees	2.2 ​± ​2.6	2.68 ​± ​2.4	1.95 ​± ​2.6	<0.001
LDFA, degrees	86.7 ​± ​1.9	87.0 ​± ​1.7	86.6 ​± ​1.9	0.009
FMA, degrees	93.3 ​± ​1.9	93.0 ​± ​1.7	93.4 ​± ​1.9	0.009
MPTA, degrees	86.0 ​± ​2.0	85.4 ​± ​2.0	86.3 ​± ​1.9	<0.001
JLCA, degrees	1.45 ​± ​1.4	1.10 ​± ​1.3	1.55 ​± ​1.5	<0.001

Data are presented as means ​± ​standard deviations.

*Abbreviations:* HKAA, hip–knee–ankle angle; LDFA, lateral distal femoral angle; FMA, femoral mechanical axis; MPTA, medial proximal tibial angle; JLCA, joint line convergence angle.

**Table 2:** The top ten knee phenotypes of the male and female participants.

Rank	Male			Female		
	phenotype	number	%	phenotype	number	%
1	VAR_HKA_6° ​+ ​NEU_FMA_0° ​+ ​VAR_MPTA_3°	149	27	NEU_HKA_0° ​+ ​VAR_FMA_6° ​+ ​NEU_MPTA_0°	208	26.2
2	VAR_HKA_6° ​+ ​NEU_FMA_0° ​+ ​NEU_MPTA_0°	135	24.5	VAR_HKA_3° ​+ ​VAR_FMA_6° ​+ ​VAR_MPTA_3°	115	14.5
3	VAR_HKA_6° ​+ ​VAL_FMA_3° ​+ ​VAR_MPTA_3°	68	12.3	VAL_HKA_3° ​+ ​VAR_FMA_6° ​+ ​NEU_MPTA_0°	109	13.7
4	VAR_HKA_6° ​+ ​VAR_FMA_3° ​+ ​NEU_MPTA_0°	44	8	NEU_HKA_0° ​+ ​VAR_FMA_6° ​+ ​VAR_MPTA_3°	92	11.6
5	VAR_HKA_6° ​+ ​VAL_FMA_3° ​+ ​NEU_MPTA_0°	41	7.4	VAL_HKA_3° ​+ ​VAR_FMA_6° ​+ ​VAL_MPTA_3°	57	7.2
6	VAR_HKA_6° ​+ ​VAR_FMA_3° ​+ ​VAR_MPTA_3°	36	6.5	VAR_HKA_3° ​+ ​VAR_FMA_3° ​+ ​NEU_MPTA_0°	43	5.4
7	VAR_HKA_6° ​+ ​NEU_FMA_0° ​+ ​VAR_MPTA_6°	28	5.1	VAR_HKA_3° ​+ ​VAR_FMA_6° ​+ ​NEU_MPTA_0°	29	3.7
8	VAR_HKA_6° ​+ ​NEU_FMA_0° ​+ ​VAL_MPTA_3°	17	3.1	VAR_HKA_6° ​+ ​VAR_FMA_3° ​+ ​VAR_MPTA_3°	19	2.4
9	VAR_HKA_6° ​+ ​VAL_FMA_3° ​+ ​VAL_MPTA_3°	9	1.6	VAR_HKA_3° ​+ ​VAR_FMA_6° ​+ ​VAR_MPTA_6°	17	2.1
10	VAR_HKA_6° ​+ ​VAR_FMA_3° ​+ ​VAL_MPTA_3°	9	1.6	NEU_HKA_0° ​+ ​VAR_FMA_3° ​+ ​VAL_MPTA_3°	15	1.9

*Abbreviations:* VAR, varus; NEU, neutral; VAL, valgus; HKA, hip–knee–ankle angle; FMA, femoral mechanical axis; MPTA, medial proximal tibial angle.

**Fig. 1:**
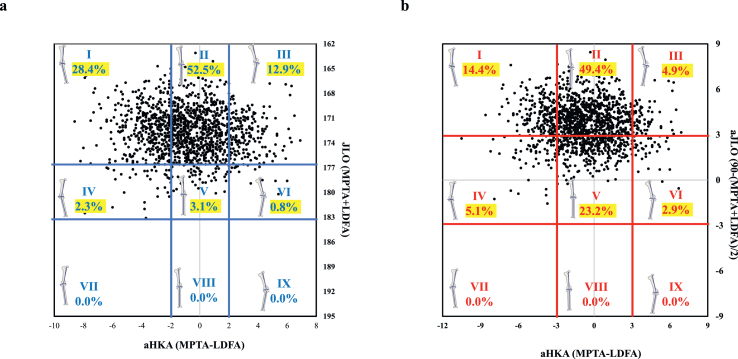
Plot of arithmetic hip–knee–ankle angle (aHKA) against joint line obliquity showing the distribution (by percentage) of the nine original CPAK types (a) and modified CPAL types (b). LDFA, lateral distal femoral angle; MPTA, medial proximal tibial angle.

The distribution of knee phenotypes in the overall cohort differed substantially between the original and modified CPAK classifications. With the original CPAK classification (Fig. 1a), most knees were clustered into types II (52.5 ​%) and I (28.4 ​%), with only 3.1 ​% of knees being categorized as type V. With the modified CPAK classification (Fig. 1b), the distribution was relatively balanced, with types II (49.4 ​%), V (23.2 ​%), and I (14.4 ​%) emerging as the predominant categories.

**Fig. 2:**
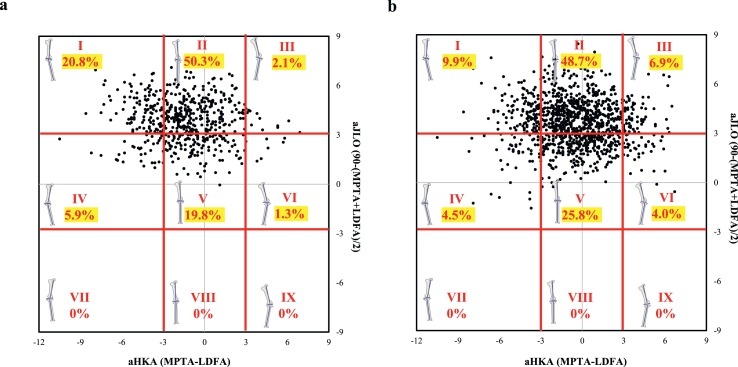
Plot of arithmetic hip–knee–ankle angle (aHKA) against aJLO showing the distribution (by percentage) of the nine modified CPAK types between men (a) and women (b). LDFA, lateral distal femoral angle; MPTA, medial proximal tibial angle.

Regarding sex-specific differences, types II (48.7 ​%) and V (25.8 ​%) were the leading categories in women, with type I accounting for 9.9 ​%. In contrast, men had a higher prevalence of type I (20.8 ​%) alongside types II (50.3 ​%) and V (19.8 ​%) (Fig. 2).

**Fig. 3:**
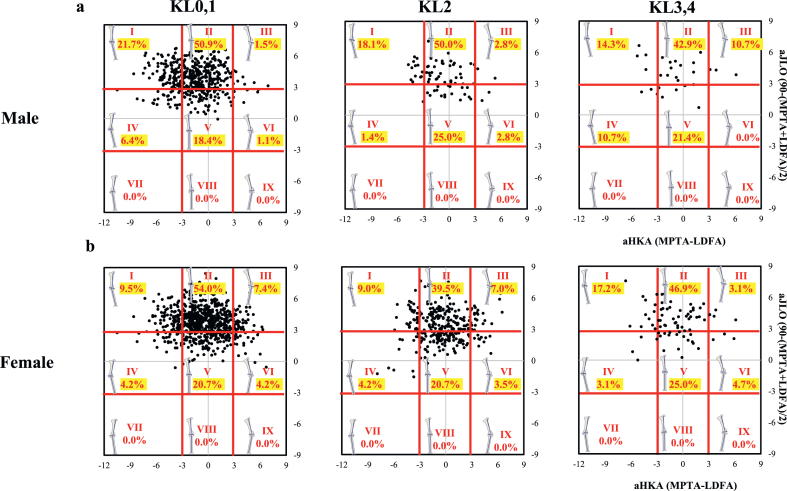
Plot of arithmetic hip–knee–ankle angle (aHKA) against aJLO showing the distribution (by percentage) of the nine modified CPAK types with Kellgren–Lawrence (KL) grades 0, 1, 2, 3, and 4. LDFA, lateral distal femoral angle; MPTA, medial proximal tibial angle.

The radiographic severity of knee OA revealed relatively stable patterns in men, with types II and V being consistently predominant across KL grades. In men, the majority of KL grade 0–1 knees were classified as types II (50.0 ​%) and V (25.0 ​%); this distribution was maintained in advanced knee OA (Fig. 3a). In women, valgus phenotypes were more frequently represented, and 11.6 ​% of KL grade 0–1 knees were classified as types III and VI; this remained evident even in KL grade 3–4 knees (Fig. 3b).

**Fig. 4:**
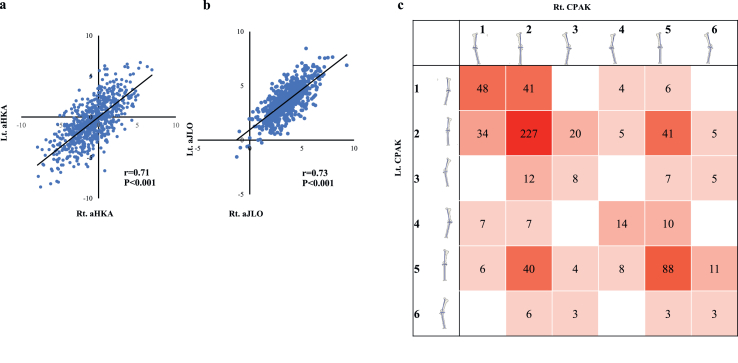
Correlation and concordance of bilateral knee alignment parameters. (a) Scatterplot showing the strong correlation of arithmetic hip–knee–ankle angle (HKAA) between right and left knees (*r* ​= ​0.71, *p* ​< ​0.001). (b) Scatterplot showing the strong correlation of aJLO between sides (*r* ​= ​0.73, *p* ​< ​0.001). (c) Heatmap analysis of bilateral CPAK classification revealed that 388 individuals (58 ​%) exhibited concordant phenotypes in both knees, whereas the remainder showed discordant phenotypes.

Analysis of side-to-side asymmetry revealed a relative strong correlation with the aHKA (*r* ​= ​0.71, *p* ​< ​0.001) (Fig. 4a) and a similarly strong correlation with the aJLO (*r* ​= ​0.73, *p* ​< ​0.001) (Fig. 4b). Heatmap analysis of CPAK categories confirmed the presence of bilateral concordance in only 388 individuals (58 ​%); the remaining knees demonstrated discordant phenotypes (Fig. 4c).

## 4 Discussion

In the present study, the CPAK classification in a large community-based cohort was investigated by comparing the original and modified CPAK classifications and analyzing sex-specific differences and side-to-side asymmetry. This study yielded important findings. First, the modified CPAK classification redistributed the knees more evenly across phenotypes and corrected the underrepresentation of neutral–neutral type V knees evident in the original CPAK classification. Second, sex-specific differences were observed, with women more frequently exhibiting neutral alignment (type V) and men showing higher proportions of varus (type I) phenotypes. Finally, side-to-side concordance was limited, with only 58 ​% of individuals showing identical modified CPAK types bilaterally.

The original CPAK classification, introduced by MacDessi et al., in 2021, provides a comprehensive framework for describing constitutional knee phenotypes by combining the aHKA and joint line obliquity [9]. The CPAK classification offers a standardized nomenclature for both healthy and OA knees by stratifying knees into nine categories according to varus, neutral, or valgus limb alignment, along with apex distal, neutral, or apex proximal joint line orientation. This system, which is specifically designed to capture pre-arthritic alignment, has been increasingly employed in surgical planning for knee arthroplasty [2,23]. Certain phenotypes have been shown to dominate across populations. In particular, a previous study reported that type II was the most prevalent in both healthy and OA cohorts, accounting for approximately 39 ​% of healthy knees and 32 ​% of OA knees, followed by types I and V [9]. Phenotypes with apex proximal joint line obliquity (types VII–IX) are extremely rare, accounting only for a lower percentage of cases. Consequently, the traditional surgical target of a neutral mechanical axis with a horizontal joint line corresponds to only a minority of constitutional phenotypes.

The application of the CPAK classification to Asian populations led to a markedly skewed distribution. Hsu et al. reported that the use of the original CPAK classification resulted in an excessive clustering of knees into apex distal types, with neutral–neutral type V accounting for <5 ​% of cases [14]. To address this limitation, they proposed the modified CPAK classification to reflect joint line orientation to the ground more accurately. The modified CPAK classification redistributed the knees more evenly across phenotypes, increasing the proportion of type V knees to approximately 25 ​%. Our results confirmed and extended the findings of Hsu et al. In our study, which included a larger population with a broad spectrum of OA severity, the proportion of type V knees likewise increased when the modified CPAK classification was applied. These findings support the notion that the modified CPAK classification is more appropriate for Asian populations, who characteristically exhibit greater constitutional varus and wider alignment variability.

Sex-specific analyses further highlighted distinct patterns. The prevalence of valgus phenotypes (types III and VI) was higher in women, whereas the proportion of varus (type I) alignment was greater in men. These findings are consistent with known anatomical and biomechanical sex-specific differences, such as wider pelvis, greater femoral anteversion, and larger Q-angle in women, all of which predispose them to valgus knee alignment [24,25]. According to previous research, women more frequently present with valgus morphology, whereas men more often demonstrate varus alignment.

A key finding of this study is the limited bilateral concordance of CPAK classification. Despite strong correlations with the aHKA and aJLO between the right and left limbs, only 58 ​% of participants had identical CPAK types bilaterally. Investigations on side-to-side asymmetry in lower-limb alignment are scarce. Beckers et al. analyzed 250 volunteers and observed symmetry in 79 ​% of cases using the HKAA classification (i.e., neutral alignment was defined for limbs with HKAA of 180° ​± ​3°, and values smaller than 177° and higher than 183° were considered varus and valgus, respectively) and in 59 ​% of cases using a phenotype-based approach [26]. They concluded that the contralateral limb could not reliably serve as an ideal reference for alignment reconstruction [26]. Sava et al. analyzed 141 patients and reported that only 26 ​% had identical coronal phenotypes bilaterally, further supporting the notion that contralateral templating is unreliable [27]. Conversely, Jacquet et al. evaluated 233 healthy individuals and reported minimal asymmetry (<2 ​%) for the HKAA, MPTA, and LDFA, suggesting near-symmetry in healthy populations [28]. Pujol et al. reported 38 ​% of CPAK concordance in patients undergoing total knee arthroplasty and cautioned against the use of the unaffected limb as a reference [29]. These discrepancies across studies may be attributable to their relatively small sample sizes and heterogeneous methodologies employed. Our analysis was conducted on a comparatively large community-based cohort, allowing for a more robust estimation of bilateral concordance in coronal knee alignment.

Recent studies have expanded the understanding of coronal alignment phenotypes beyond static classifications such as CPAK by examining their behavior under different surgical alignment philosophies [30,31]. Franceschetti et al. [32] showed that mechanically aligned total knee arthroplasty yields heterogeneous outcomes across CPAK phenotypes, while Graichen et al. [33] reported that tibia-first, gap-balanced, patient-specific alignment can restore native bony phenotypes and joint line orientation in most cases. Recent investigations have also highlighted the conceptual and functional limitations of CPAK. Critical appraisals and intraoperative analyses have demonstrated that the HKA axis changes dynamically during motion and that static classifications such as CPAK may oversimplify true knee kinematics [34,35]. Integrating morphological frameworks such as CPAK with dynamic and functional assessment tools may therefore provide a more comprehensive understanding of coronal plane behavior and facilitate more individualized alignment evaluation in future research.

This study has some limitations. First, the cross-sectional design precluded longitudinal evaluation of CPAK phenotype changes over time. Although the classification system has been reported to remain relatively stable during OA progression, further longitudinal studies should be conducted to confirm phenotype stability in populations. Second, small differences in positioning may affect angular measurements; however, radiographic acquisition was standardized in this study. Third, as this was a single-center study, selection bias cannot be completely excluded. Finally, conceptual limitations of the CPAK classification should be acknowledged. The CPAK system represents a simplified, static framework of coronal alignment that does not account for dynamic joint line changes during knee motion [36]. Moreover, although nine theoretical CPAK categories exist, population-based studies have shown that only six are typically observed, reflecting the system’s limited ability to capture the full spectrum of functional knee phenotypes. Future research integrating CPAK with three-dimensional and motion-based analyses may provide a more comprehensive understanding of coronal alignment.

In conclusion, the modified CPAK classification provides a more balanced and accurate representation of coronal alignment in a Japanese population, consistent with previous findings from other Asian cohorts. Sex-specific differences and frequent bilateral asymmetry emphasize the need for individualized assessment of coronal alignment. These findings offer valuable reference data for interpreting alignment variations in clinical and epidemiological settings and may assist in the diagnosis and monitoring of early or progressive knee OA. Future research should investigate the longitudinal stability of CPAK phenotypes and their relationship to dynamic or functional alignment concepts. Future research should investigate the longitudinal stability of CPAK phenotypes and explore how these static morphological classifications relate to dynamic or functional alignment concepts in knee arthroplasty.

## Author contributions

KI was responsible for the organization and coordination of this study, and was the chief investigator and responsible for the data analysis. RK, ES, HKI, YS, YK, ET and developed this study design. All authors contributed to the writing of the final manuscript.

## Data availability statement

The data sets generated during and/or analyzed during the current study are available from the corresponding author on reasonable request.

## Declaration of generative AI and AI-assisted technologies in the writing process

N/A.

## Role of the funding source

The funding agencies (JST COI, JSPS, Health Labor Sciences Research Grant, Japanese Orthopaedic Association, and the Japan Orthopaedics and Traumatology Research Foundation) had no role in the design of the study, the collection, analysis, and interpretation of the data, the writing of the manuscript, or the decision to submit the article for publication. Their support was limited to financial assistance.

## Conflict of interest

The authors declare no conflicts of interest.
